# Relationship between Nutritional Screening Tools and GLIM in Complicated IBD Requiring Surgery

**DOI:** 10.3390/nu13113899

**Published:** 2021-10-30

**Authors:** Camilla Fiorindi, Gabriele Dragoni, Stefano Scaringi, Fabio Staderini, Anita Nannoni, Ferdinando Ficari, Francesco Giudici

**Affiliations:** 1 Department of Health Science, Careggi University Hospital, 50134 Florence, Italy; camilla.fiorindi@unifi.it (C.F.); nannonia@aou-careggi.toscana.it (A.N.); 2 Department of Experimental and Clinical Biomedical Sciences “Mario Serio”, University of Florence, 50134 Florence, Italy; gabriele.dragoni@unifi.it; 3 Department of Experimental and Clinical Medicine, University of Florence, 50134 Florence, Italy; stefano.scaringi@unifi.it (S.S.); fabio.staderini@unifi.it (F.S.); ferdinando.ficari@unifi.it (F.F.)

**Keywords:** IBD, GLIM, surgery, nutritional screening tool, malnutrition

## Abstract

Background: Accurate identification of malnutrition and preoperative nutritional care in Inflammatory Bowel Disease (IBD) surgery is mandatory. There is no validated nutritional screening tool for IBD patients. We developed a novel nutritional screening tool for IBD patients requiring surgery and compared it with other tools. Methods: we included 62 consecutive patients scheduled for elective surgery. The IBD Nutritional Screening tool (NS-IBD) was developed to screen patients for further comprehensive assessment. NRS-2002, MUST, MST, MIRT, SaskIBD-NR are compared with the new test. All screening tests were subsequently related to new GLIM criteria. Results: according to GLIM criteria, 25 (40%) IBD patients were malnourished (15 CD and 10 UC, 33% vs. 63%, *p* = 0.036). Stage 1 malnutrition was reported in ten patients, while stage 2 was detected in 15 patients. The comparison of each nutritional risk tool with GLIM criteria showed sensitivity of 0.52, 0.6, 0.6, 0.84, 0.84 and 0.92 for SASKIBD-NR, MUST, MST, NRS-2002, MIRT, and the new NS-IBD, respectively. Conclusions: in IBD, currently adopted nutritional screening tools are characterized by a low sensitivity when malnutrition diagnosis is performed with recent GLIM criteria. Our proposed tool to detect malnutrition performed the best in detecting patients that may require nutritional assessment and preoperative intervention.

## 1. Introduction

Nutritional screening tools are commonly used in clinical practice to identify patients at risk of malnutrition. Patients at nutritional risk must receive more comprehensive assessments to establish malnutrition diagnosis, thus providing the basis for individualised treatment plans. The ESPEN guidelines for clinical nutrition in inflammatory bowel diseases (IBD) state that IBD patients are particularly at risk of malnutrition, recommending to screen for malnutrition at the time of diagnosis and then regularly during follow-up [1]. Malnutrition in both Crohn’s disease (CD) and Ulcerative Colitis (UC) worsens the prognosis and the quality of life, increasing the rate of complications and mortality [1]. Among malnutrition screening tools, Nutritional Risk Screening 2002 (NRS-2002), Malnutrition Universal Screening Tool (MUST), Malnutrition Screening Tool (MST) as well as IBD-specific tests, such as Malnutrition Inflammation Risk Tool (MIRT) and the Saskatchewan IBD–Nutrition Risk (SaskIBD-NR), are the most frequently used [2]. A recent systematic review on the screening and assessment of malnutrition in IBD concluded that there is a high heterogeneity between the available nutrition screening tools, implying that we are far from having an accurate risk detection [3]. Furthermore, the aetiology of malnutrition in IBD is multifactorial as it depends on the combination of inflammatory response, clinical complications, medical therapies, and surgical treatment; inflammation, strictures, abscesses, fistulas, and previous surgical resections may be responsible for decreased intake, nutrient losses and malabsorption [4,5]. Actually, a validated nutritional screening tool specific for IBD patients is still lacking. In fact, the IBD specific screening tests available were created based on expert opinion and literature findings without any validation process. Recently, the Global Leadership Initiative on Malnutrition (GLIM) involved the major clinical nutrition Societies to reach a global consensus on the identification of accurate criteria for the diagnosis of malnutrition in clinical settings [6]. After the fully validation of GLIM, it seems necessary to adopt a malnutrition screening tool that includes phenotypical and etiological parameters [7].

The aim of this study is to evaluate, in an IBD setting, the presence of malnutrition according to the recent GLIM criteria. In addition, in accordance with the adoption of these criteria, we created a new screening tool for the initial evaluation of IBD patients (NS-IBD). This new specific malnutrition screening test adds the classic parameters to the peculiar characteristics of IBD to clearly identify patients who can benefit from a nutritional treatment. The novel screening test was compared with the available screening tools NRS-2002, MUST, MST, MIRT and SaskIBD-NR to assess their concordance. The ability of each screening test performed to detect malnutrition according to GLIM was analysed.

## 2. Materials and Methods

### 2.1. Study Population and Design

Prospectively, consecutive patients affected by complicated IBD and scheduled for elective surgery at Careggi University Hospital in Florence between December 2018 and March 2020 were included in the study. After obtaining Ethical approval by a Local Expert Scientific Committee, each patient was screened for nutritional risk adopting the most used tools in adults, such as NRS-2002 [8], MUST [9], MST [10], and in IBD patients, such as MIRT [11] and SaskIBD-NR [12] (Table 1). The mean time of nutritional evaluation after being placed in the surgical waiting list was 12 ± 8 days.

C-Reactive Protein (CRP); Gastrointestinal (GI); Nutritional Risk Screening 2002 (NRS-2002); Malnutrition Universal Screening Tool (MUST); Malnutrition Screening Tool (MST); Saskatchewan IBD–Nutrition Risk (SaskIBD-NR); Malnutrition Inflammation Risk Tool (MIRT)

We recorded the prevalence of high nutritional risk resulting from each screening test and analysed their validity by comparing them with the new proposed GLIM criteria for malnutrition diagnosis [6] evaluated during the same outpatient nutritional visit.

This aspect was also analysed in relation to the severity of malnutrition in Stage 1 (moderate) and Stage 2 (severe), according to GLIM criteria. All patients received comprehensive nutritional assessment, including anthropometric parameters [body weight, height, body mass index (BMI), unintended weight loss (UWL)]. Food and nutrition related history were calculated by the average of the 3-day patient reported intakes using WINFOOD software (Pro 3.15.x version; Medimatica, Teramo, Italy), and body composition through bio-impedance vector analysis (BIVA) calibrated device (Nutrilab-Monitor, AKERN, Florence, Italy) analysed with Bodygram™ software. The total energy expenditure (TEE) was calculated according to ESPEN guidelines for clinical nutrition in IBD [1]. A new IBD-specific nutritional screening tool (NS-IBD) consistent with GLIM criteria was developed and tested in all patients. It was developed (Table 2) considering the nutritional parameters reported to be specific for IBD patients, and adopting as cut-off values of each parameter the same used by GLIM criteria, as follows:

(1) Anthropometric parameters (consistent with GLIM phenotypic criteria)
  - BMI: values under <18.5 kg/m^2^ are associated with poor outcome and higher mortality rates [13,14]. GLIM BMI cut-off for malnutrition risk is <20 kg/m^2^. In older adults, the cut-off for the definition of underweight is higher (<22 kg/m^2^) as carrying some extra weight seems to be protective in this population [6,15];
  - UWL: it is reported to be associated with high morbidity and mortality rates as indirect sign of catabolic status [16]. In particular, UWL >5% within the last 6 months, or >10% beyond the last 6 months were considered prognostic for malnutrition [6];
(2) Disease-related parameters (consistent with GLIM etiologic criteria)
  - Chronic diarrhoea, ileostomy and previous surgery for IBD: many studies showed that all these three parameters are associated with malnutrition or body weight loss [17,18,19,20,21,22,23];
  - Other gastrointestinal (GI) symptoms (nausea, vomiting, bloating, diarrhoea, abdominal pain and decreased appetite): it is well known that reduced absorption of food/nutrients is associated with the occurrence of these symptoms [6,19,23,24].

### 2.2. Statistical Analysis

Descriptive statistics of nominal data were described with raw numbers and percentages, while continuous variables were reported with mean and standard deviation (SD). Categorical variables were analysed using Fisher’s exact test or Pearson’s χ^2^ test and continuous variables were analysed using Student’s t-test or Mann–Whitney’s *u*-test, as appropriate, with a statistically significant association when *p* < 0.05. For the evaluation of reliability of the different malnutrition screening tools compared to malnutrition diagnosis according to GLIM criteria we calculated sensitivity, specificity, positive predictive value (PPV), negative predictive value (NPV) and Youden Index for each test, and the Receiver Operating Characteristic (ROC) analysis for NS-IBD. The relationship between nutritional risk and postoperative length of stay was evaluated adopting Pearson’s χ^2^ test. Cohen’s kappa statistic was performed to measure the agreement between all screening tests completed. Statistical analysis was performed with Origin-Pro software (OriginLab Corporation, Northampton, MA 01060, USA), version 2020b.

## 3. Results

### 3.1. Demographics and Clinical Characteristics of IBD Patients

Sixty-two IBD patients were included, 46 CD (74%) and 16 UC (26%), with a mean age of 51.4 years (20–79). The mean duration of disease was 12.5 years. In CD group, 18 patients (39%) were scheduled for operation due to surgical recurrence, while 28 patients (61%) were at their first abdominal surgery. Thirty-six CD patients (78%) had an ileal disease localization, 5 patients (11%) an ileocolonic localization and 5 (11%) an isolated colonic disease. A stricturing CD was present in 31 patients (67%), fistulizing behaviour in 11 patients (24%) and inflammatory in 4 (9%). Of the 16 UC patients, 5 (31%) were scheduled for first-step surgery, whereas 10 of the remaining 11 were scheduled for second-step surgery after previous subtotal colectomy and 1 had been scheduled for total proctocolectomy after a previous right hemicolectomy for T1 adenocarcinoma. The presence of ileostomy was more prevalent in UC than CD (63% vs. 2%).

### 3.2. Nutritional Characteristics of IBD Patients

Sixteen CD patients (35%) had chronic diarrhoea. More than 3 GI symptoms were reported by 7 CD patients (15%), while all UC patients declared 2 or less GI symptoms. The most frequent GI symptoms were nausea (8%), vomiting (3%), bloating/abdominal pain (61%) and decreased appetite (15%).

Four CD (9%) and 4 UC patients (25%) were underweight. Overweight and obesity were present in 10 (22%) and 2 (4%) CD patients, respectively, and in 1 (6%) and 1 (6%) UC patients, respectively. Twenty-seven CD patients (29%) and 5 UC patients (31%) did not lose weight in the previous year. An UWL of more than 10% of the usual weight was present in 6 CD (13%) and 4 (25%) UC patients. Globally, 37% of IBD patients showed an UWL more than 5% during the 6 months before our assessment.

Regarding Free Fat Mass (FFM), the mean value was slightly higher in CD (79.1%) than in UC patients (77.4%). The mean value of FFM index (FFMI) was 17.7 kg/m^2^ and 17.2 kg/m^2^ in CD and UC patients, respectively. FFMI values consistent with sarcopenia were detected in 15 IBD patients (24%), of whom 9 CD (19.5%) and 6 UC (37.5%); ten of them had experienced UWL > 5% during the previous 3–6 months, while 8/15 were underweight and 3/15 had normal BMI with no UWL. Twelve IBD patients (19%) (9 CD and 3 UC) reported a reduction of food intake, but only 3 CD patients had an energy intake <75% of the total energy expenditure. In Table 3, baseline and nutritional characteristics of our IBD cohort are summarised.

The presence of ileostomy was associated with a significantly lower FFMI (16.30 vs. 17.84, kg/m^2^) (*p* = 0.037), and a higher rate of UWL (82% vs. 43%) (*p* = 0.046). A significantly lower BMI (20.70 kg/m^2^ vs. 23.62 kg/m^2^) (*p* = 0.005) and FFMI (16.67 kg/m^2^ vs. 18.36, kg/m^2^) (*p* = 0.002) were also found in patients with previous IBD surgery compared to patients at first operation.

Patients with ≥3 symptoms were reported to have numerically higher values of white blood cells (10.7 × 10^9^/L vs. 8.7 × 10^9^/L) (*p* = 0.063), fibrinogen (441 mg/dL vs. 373 mg/dL) (*p* = 0.066) and C-reactive protein (39 mg/L vs. 27 mg/L) (*p* = 0.762).

### 3.3. Prevalence of Nutritional Risk

**Figure 1:**
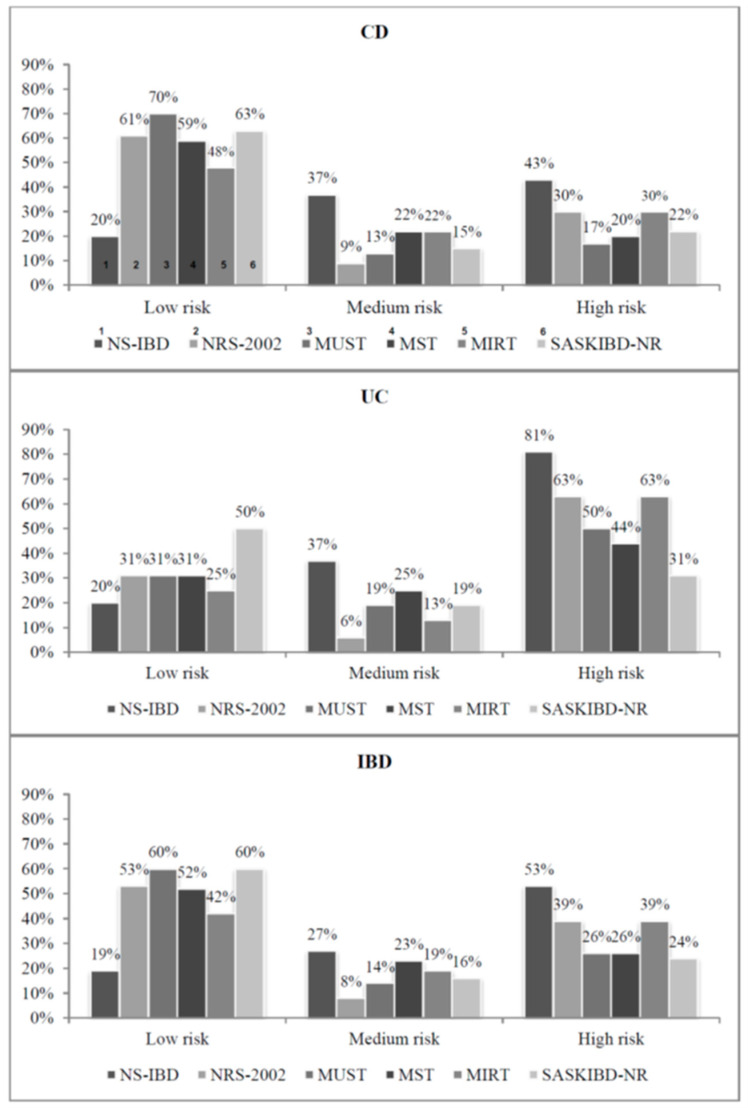
Nutritional screening results in our IBD cohort.

Depending on the different nutritional risk tool tested, the prevalence of high nutritional risk ranged from 24% to 53%. The SASKIBD-NR, the MUST and the MST showed the lowest prevalence of patients with higher risk of malnutrition (24%, 26% and 26%, respectively), while the NS-IBD detected the highest (53%). According to the NRS-2002 and the MIRT, the prevalence of high nutritional risk was 39%. The results of the several screening tools differed also in case of medium and low nutritional risk (Figure 1). UC patients were at higher nutritional risk than CD patients, with overall agreement for each nutritional risk that was used. Only the MST and the SASKIBD-NR did not report a significant difference between UC and CD groups.

### 3.4. Screening Tests Agreement

NS-IBD had a good Cohen’s kappa concordance only with NRS-2002 (k = 0.650). While the comparisons with all the other tools showed only moderate agreement (k < 0.6).

### 3.5. Reliability of the NS-IBD and Other Screening Tests with GLIM Malnutrition Diagnosis

With regard to malnutrition diagnosis according to GLIM criteria, 25 IBD patients (40%) resulted malnourished (15 CD and 10 UC, 33% vs. 63%, *p* = 0.036). Particularly, stage 1 malnutrition was present in 10 patients (7 CD and 3 UC), whereas stage 2 was detected in 15 patients (8 CD and 7 UC). Based on previous ESPEN 2015 criteria, malnutrition was diagnosed in 15 IBD patients (24%), of whom 8 CD (17%) and 7 UC (44%) (*p* = 0.034) (Table 4).

The comparison of each nutritional risk tool with GLIM criteria, showed that NS-IBD was performing the best in terms of sensitivity (0.92), whereas the SASKIBD-NR (0.52), the MUST and the MST (0.6) were the least sensitive. The NRS-2002 and the MIRT had a sensitivity of 0.84. The tools with the highest specificity were the MUST (0.97) and the MST (0.97), while the NS-IBD had a specificity of 0.73 The NRS-2002, the MIRT and the SASKIBD-NR showed specificity of 0.92, 0.92 and 0.95, respectively. Youden Index is calculated for each screening test (Table 5).

**Figure 2:**
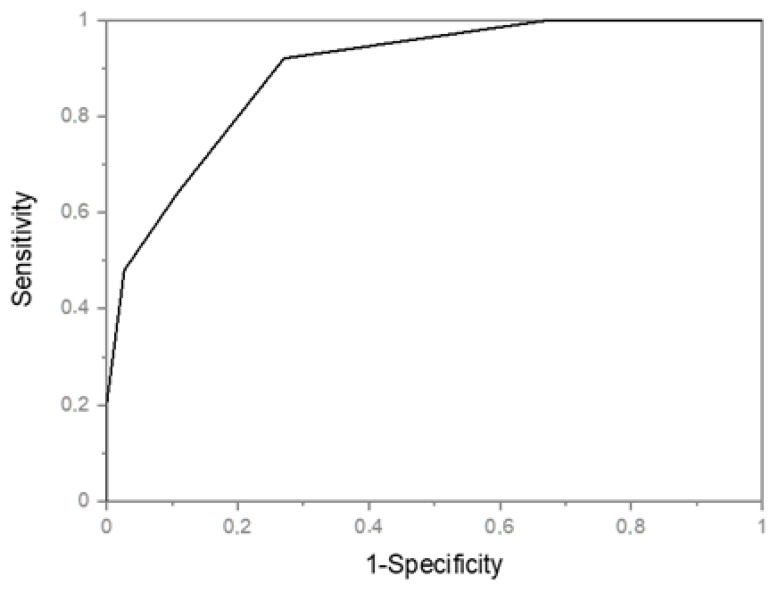
NS-IBD ROC Curve. IBD Nutritional Screening tool (NS-IBD); Receiver Operating Characteristic (ROC).

The calculated area under the ROC curve of NS-IBD test in relationship to GLIM showed a good accuracy (0.89459, *p* < 0.0001) (Figure 2).

### 3.6. Postoperative Length of Stay and Nutritional Risk

**Figure 3:**
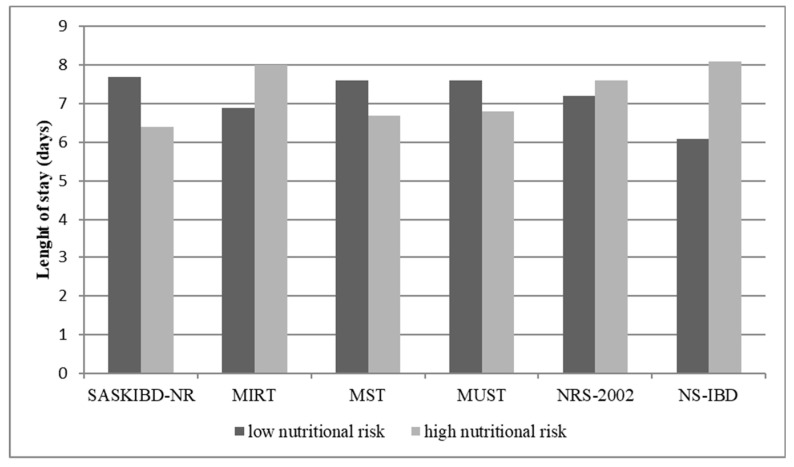
Nutritional screening results in relationship to the length of stay.

Assessing the relationship between the malnutrition risk and the postoperative length of stay (LOS) we found that according to NS-IBD, the mean LOS of patients with low nutritional risk was 6.1 days (±1.5), while the mean LOS of patients with high nutritional risk was 8.1 days (±5.9) (*p* = 0.098). NS-IBD resulted the most accurate tools in predicting LOS (Figure 3).

Inflammatory bowel disease (IBD); Crohn’s disease (CD); Ulcerative colitis (UC); IBD Nutritional Screening tool (NS-IBD); Nutritional Risk Screening 2002 (NRS-2002); Malnutrition Universal Screening Tool (MUST); Malnutrition Screening Tool (MST); Malnutrition Inflammation Risk Tool (MIRT); Saskatchewan IBD–Nutrition Risk (SaskIBD-NR)

IBD Nutritional Screening tool (NS-IBD); Nutritional Risk Screening 2002 (NRS-2002); Malnutrition Universal Screening Tool (MUST); Malnutrition Screening Tool (MST); Malnutrition Inflammation Risk Tool (MIRT); Saskatchewan IBD–Nutrition Risk (SaskIBD-NR)

## 4. Discussion

In hospitalised patients, the commonly adopted nutritional screening tools are quite sensitive (60–100%) [25,26], but a particular focus on IBD patients is currently lacking. Furthermore, sensitivity of the previously reported tools in relation to the recent GLIM criteria for malnutrition diagnosis has not yet been investigated. Our experience with 62 IBD patients showed a lower sensitivity (range 52–84%) of the conventional nutritional screening tools when performing malnutrition diagnosis adopting GLIM criteria. Differentially, according to GLIM, our new developed tool NS-IBD has a sensitivity of 92% and specificity of 73%, with 0.7 of positive predictive value and 0.93 of negative predictive value and Youden Index of 0.65. In oncological patients that are candidate for elective surgery in Enhanced Recovery After Surgery (ERAS) setting, a timely preoperative nutritional intervention has revealed to be fundamental in influencing the short-term outcome [27]. It is well known that both IBD surgery is characterized by a higher incidence of postoperative complications [28,29] and malnutrition is a major risk factor [30]. With that in mind, it is clear that the development of a highly sensitive nutritional screening tool is necessary for IBD patients requiring surgical treatment to properly correct their malnutrition status, minimise the risk of postoperative complications and subsequently reduce the hospital stay and the costs for the healthcare system.

The parameters we included in the NS-IBD were BMI, UWL, previous abdominal IBD surgery, presence of chronic diarrhoea or ileostomy, and presence of specific gastrointestinal symptoms (nausea, vomiting, bloating, abdominal pain and decreased appetite). The BMI may be biased by fluid overload and oedemas and does not accurately describe body composition. In IBD, malabsorption seems to play a major role in patients with BMI less than 18.5 kg/m^2^ [31]. Our patients had a mean BMI of 22.9, and only 13% were underweight. In fact, the BMI alone does not reflect potentially pathological weight losses or the actual food intake. Thus, UWL is included in the majority of nutritional screening tools as it indirectly reveals a decreased FFM [16]. FFM can be estimated with BIVA, thoracic CT scan or Dual X-ray Absorptiometry (DXA), but all these tests are usually absent in real-life initial nutritional evaluation. IBD patients develop a relative reduction in FFM and increase in adiposity over time. This may occur due to chronically poor dietary intake, increased rates of protein turnover and gut loss of nutrients during flares of active disease or the effect of disease treatments [32]. Before surgery, weight loss is associated with an increased risk of severe surgical complications [33]. In our cohort, 48% experienced UWL with a surprisingly higher frequency in UC than in CD (61% vs. 42%, respectively).

In regard to previous abdominal IBD surgery, a nationwide study performed in an IBD setting found that previously resected patients had higher risks of malnutrition or UWL [17,18]. A multivariate analysis revealed that the variables associated with a higher risk of malnutrition were history of abdominal surgery, due to continuous clinical activity, and avoidance of some food groups during a flare [OR = 10.3, 95% CI = 1.3–78.1] [19]. We found malnutrition according to GLIM criteria in 52% of IBD patients with previous abdominal resective surgery. Particularly, 73% of patients with Stage 2 malnutrition according to GLIM had previous surgery for IBD (*p* = 0.035).

Ongoing and severe diarrhoea, or ileostomy, can result in malabsorption with subsequent UWL, malnutrition, nutritional deficiencies and dehydration [20]. Malabsorption is the predominant contributing factor to malnutrition in IBD [21,23].

We found a significantly higher prevalence of malnutrition according to GLIM in patients with ileostomy or chronic diarrhoea (*p* = 0.021), with 47% of all patients with GLIM Stage 2 malnutrition having an ileostomy (*p* = 0.002). Our study showed that the presence of ileostomy was significantly associated with lower FFMI values and higher rate of UWL, whereas the history of previous IBD surgery was significantly associated with lower BMI and FFMI values. The assessment of body composition, particularly of FFM, is carried out with the use of one of the several available methods (BIVA, DXA, CT), which makes the inclusion of FFM value in malnutrition screening tools challenging, due to their heterogeneity. The new NS-IBD includes the presence of ileostomy and previous IBD surgery as nutritional risk parameters, to identify patients at higher risk of muscle mass depletion, even intentionally omitting FFM measurements which require experienced figures and specific devices to be calculated.

The presence of specific gastrointestinal symptoms, such as nausea, vomiting, bloating, abdominal pain and decreased appetite, have been incorporated as supportive indicators into GLIM consensus, as they can indirectly reveal the presence of etiological criteria [6]. The decreased appetite and the subsequent reduced oral food intake are important reasons for malnutrition in patients affected with IBD. Two main mechanisms are reported: the first is connected to the disease itself, as patients avoid eating due to symptoms such as nausea, abdominal pain, vomiting, and diarrhoea during disease flare [23,24]; the second is due to food intake self-restriction to prevent or treat a flare, both are associated with a higher risk of malnutrition [19].

Blood tests have been intentionally left out of NS-IBD to avoid biochemical examination to be part of the nutritional screening. We believe it is necessary to be able to easily and timely use the tool, if possible, at the preliminary discussion of the case, during the multidisciplinary IBD meeting when surgery is proposed. Furthermore, patients with ≥3 symptoms were found to have higher values of white blood cells and fibrinogen, which might influence the tool reliability. Laboratory values are mostly delayed and costly. Additionally, numerous non-nutrition-related factors may influence the laboratory parameters [34].

Moreover, NS-IBD does not take age into consideration, and this was corroborated by our finding that age at assessment and GLIM malnutrition diagnosis were not significantly associated in our cohort.

Concerning the relationship between the different nutritional screening tools and clinical outcomes, we interestingly found that the nutritional risk evaluated with NS-IBD better predicted the LOS. We were not able to statistically correlate the nutritional risk with the postoperative complications since in our cohort we had an extremely low incidence of medical and surgical complications, maybe because of the strict nutritional risk assessment and the tailored preoperative nutritional intervention. The main limitation of our study is represented by the small sample size. Validation of this new screening tool with a larger cohort of pre-surgical IBD is desirable.

## 5. Conclusions

In conclusion, we may evaluate currently adopted nutritional screening tools that are characterized by low sensitivity when malnutrition diagnosis is performed with recent GLIM criteria in IBD patients. However, NS-IBD is still a non-validated tool, as are MIRT and Sask-IBD, while NRS 2002, MUST and MST are not validated in IBD [3,11]. Regardless, patients are at a high risk of both malnutrition and the incidence of postoperative complications if nutritional status is not timely correct. Therefore, the development of a new and more sensitive screening tool seems necessary. We tested a simple IBD-specific tool able to maximize sensitivity, identifying in a simple manner, and without the need for blood or other complex exams, all patients requiring further nutritional assessment and intervention. We believe NS-IBD could be easily adopted at every outpatient visit during the preoperative course of IBD patients, and do not necessarily need to be performed only by specialized nutritionists. The mean time to perform the test in our experience was 3 min.

## Figures and Tables

**Table 1:** Mostly adopted nutritional risk screening tools.

	BMI		Weight Loss		Reduced Food Intake		Severity of Disease		CRP		GI Symptoms **		AGE
**NRS-2002**	Score 1	x	Score 1	>5% in 3 months	Score 1	Intake of 50–75% of normal requirement in preceding week	Score 1	Hip fracture, chronic patients, in particular with acute complications: cirrhosis, COPD. chronic hemodialysis, diabetes, oncology					≥70: + 1 point
	Score 2	18.5–20.5	Score 2	>5% in 2 months	Score 2	Intake of 25–60% of normal requirement in preceding week	Score 2	Major abdominal surgery, stroke, severe pneumonia, hematologic malignancy					
	Score 3	<18.5	Score 3	>5% in 1 month or >15% in 3 months	Score 3	Intake of 0–25 of normal requirement in preceding week	Score 3	Head injury, bone marrow transplantation, intensive care patients.					
**MUST**	Score 1	18.5–20	Score 1	5–10% in 3–6 months	Score 2	There has been or is likely to be no nutritional intake for >5 days OR the patients is acutely ill *							
	Score 2	<18.5	Score 2	>10% in 3–6 months									
**MST**			Score 1	1–5 kg	Score 1	Patient been eating poorly because of a decreased appetite							
			Score 2	6–10 kg									
			Score 3	5–11 kg									
			Score 4	>15 kg									
			Score 2	Unsure									
**SASKIBD-NR**			Score 1	2.3–4.5 kg in 1 month	Score 2	Eating poorly because of a decreased appetite					Score 1	N° 1–2	
			Score 2	4.5–7 kg in 1 month	Score 2	Restricting any foods or food groups					Score 2	N° ≥ 3	
			Score 3	>7 kg in 1 month									
**MIRT**	Score 1	18.5–20	Score 2	5–10% in 3 months					Score 2	5–50 mg/L			
	Score 2	<18.5	Score 3	≥10% in 3 months					Score 3	≥50 mg/L			

* Such patients include those who are critically ill, those who have swallowing difficulties (e.g., after stroke), or head injuries or are undergoing gastrointestinal surgery. ** nausea, vomiting, diarrhoea or poor appetite for greater than two weeks.

**Table 2:** The new specific for IBD nutritional screening tool (NS-IBD).

	Score 0	Score 1	Score 2
BMI, kg/m^2^	>20.5 or 22 if >70 years	18.5–20.5 or 20–22 if >70 years	<18.5 or <20 if >70 years
UWL, %	<5% within past 6 mths	5–10% within past 3–6 mths or >10% beyond 6 mths	5–10% within last mth or ≥10% in 3–6 mths
Chronic diarrhea or ileostomy	no	yes	/
Other GI symptoms, n°	0–2	≥3	/
Previuos surgery for IBD	no	yes	/
Total score: 0 = low risk; 1 = medium risk; ≥2 high risk			

Body Mass Index (BMI); Unintended Weight Loss (UWL); Gastrointestinal (GI); Inflammatory Bowel Disease (IBD).

**Table 3:** Baseline characteristics of IBD patients.

	IBD	CD	UC	*p* *
**Patients, *n* (%)**	62	46 (74%)	16 (26%)	
**Age, years, median (IQR)**	54.5 (42.3–62.8)	53.5 (43–62.2)	57 (35.7–62.2)	0.51952
**Male, *n* (%)**	36 (58%)	25 (54%)	11 (69%)	0.31461
**Duration of disease, years, median (IQR)**	10.5 (2.2–18)	10.5 (2.2–10.5)	11 (2.5–18.2)	0.92241
**First operation, *n* (%)**	33 (53%)	28 (61%)	5 (31%)	0.04083
**Presence of stoma, *n* (%)**	11 (18%)	1 (2%)	10 (63%)	<0.001
**Chronic diarrhoea, *n* (%)**	18 (29%)	16 (35%)	2 (13%)	0.11746
**N° of GI symptoms, mean, SD**	0.98 ± 1.13	1.21 ± 1.17	0.31 ± 0.70	<0.001
<3, *n* (%)	55 (89%)	39 (85%)	16 (100%)	0.17498
≥3, *n* (%)	7 (11%)	7 (15%)	0	
**Crohn’s Disease behaviour**				
-stricturing, *n* (%)		31 (67%)		
-fistulizing, *n* (%)		11 (24%)		
-inflammatory		4 (9%)		
**Crohn’s Disease’s localization**				
-Ileal, *n* (%)		36 (78%)		
-Ileocolonic, *n* (%)		5 (11%)		
-Colonic, *n* (%)		5 (11%)		
**UC, *n* (%)**				
-Proctitis			8 (50%)	
-Left side colitis			1 (6%)	
-Extensive colitis			7 (44%)	
**Postoperative complications**				0.501
-Anastomotic Leak	3 (5%)	2 (4%)	1 (6%)	
-Wound infection	1 (2%)	0	1 (6%)	
-PONV	5 (8%)	5 (11%)	0	
**Nutritional Status**				
Weight, kg, mean, SD	64.4 ± 13.75	64.1 ± 13.04	65.4 ± 16.05	0.74574
BMI, kg/m^2^, mean, SD	22.9 ± 4.03	22.9 ± 3.74	22.7 ± 4.90	0.6582
<18.5, *n* (%)	8 (13%)	4 (9%)	4 (25%)	0.18724
18.5–25, *n* (%)	40 (65%)	30 (65%)	10 (62%)	0.84486
25–30, *n* (%)	11 (18%)	10 (22%)	1 (6%)	0.26074
>30, *n* (%)	3 (5%)	2 (4%)	1 (6%)	1
**UWL**				
*n* (%)	30 (48%)	19 (41%)	11 (69%)	0.08269
<5%, *n* (%)	7 (11%)	6 (13%)	1 (6%)	0.66553
5–10%, *n* (%)	13 (21%)	7 (15%)	6 (38%)	0.0791
≥10%, *n* (%)	10 (16%)	6 (13%)	4 (25%)	0.26605
**FFM %, mean, SD**	78.7 ± 8.22	79.1 ± 8.13	77.4 ± 8.62	0.46595
**FFM % (M), mean, SD**	80.9 ± 7.84	82.1 ± 7.56	78.3 ± 8.17	0.18269
**FFM % (F), mean, SD**	75.3 ± 7.76	75.3 ± 7.36	75.2 ± 10.18	0.9771
**FFMI, kg/m^2^, mean, SD**	17.5 ± 2.22	17.7 ± 2.21	17.2 ± 2.29	0.41296
**FFMI (M), kg/m^2^, mean, SD**	18.5 ± 2.20	18.7 ± 2.27	18.1 ± 2.07	0.47845
**FFMI (F), kg/m^2^, mean, SD**	16.1 ± 1.34	16.4 ± 1.13	15.0 ± 0.85	0.0355
**FFMI < 17 (M) or < 15 (F), *n* (%)**	15 (25%)	9 (20%)	6 (37.5%)	0.16266
**Reduced food intake, *n* (%)**	12 (19%)	9 (19%)	3 (19%)	1
Intake > 75% of TEE, *n* (%)	59 (95%)	43 (94%)	16 (100%)	
Intake < 75% of TEE, *n* (%)	3 (5%)	3 (6%)	0	

Gastrointestinal (GI); Body Mass Index (BMI); Unintended Weight Loss (UWL); Free Fat Mass (FFM); Free Fat Mass Index (FFMI), * = *p* < 0.05 is statistically significant.

**Table 4:** Prevalence of high nutritional risk and malnutrition diagnosis in IBD, CD and UC patients.

	IBD		CD		UC		*p* *
**Nutritional screening tools**	n°	%	n°	%	n°	%	
NS-IBD	33	53	20	43	13	81	**0.01051**
NRS-2002	24	39	14	30	10	63	**0.02332**
MUST	16	26	8	17	8	50	**0.01024**
MST	16	26	9	20	7	44	0.05687
MIRT	24	39	14	30	10	63	**0.02332**
SASKIBD-NR	15	24	10	22	5	31	0.44417
**Malnutrition diagnosis**	n°	%	n°	%	n°	%	
GLIM	25	40	15	33	10	63	**0.03578**
- GLIM stage 1	10	16	7	15	3	19	0.70878
- GLIM stage 2	15	24	8	17	7	44	**0.03395**

Inflammatory bowel disease (IBD), Crohn’s disease (CD), Ulcerative colitis (UC); Nutritional Screening tool (NS-IBD); Nutritional Risk Screening 2002 (NRS-2002); Malnutrition Universal Screening Tool (MUST); Malnutrition Screening Tool (MST), Malnutrition Inflammation Risk Tool (MIRT); Saskatchewan IBD–Nutrition Risk (SaskIBD-NR); Global Leadership Initiative on Malnutrition (GLIM), * = *p* < 0.05 is statistically significant.

**Table 5:** Prevalence of high nutritional risk and malnutrition diagnosis in IBD, CD and UC patients.

**IBD**									
	Sensitivity	95% CI	Specificity	95% CI	PPV	95% CI	NPV	95% CI	Youden index
NS-IBD	0.92	0.72–0.98	0.73	0.56–0.86	0.7	0.51–0.84	0.93	0.76–0.99	0.65
NRS-2002	0.84	0.63–0.95	0.92	0.77–0.98	0.87	0.66–0.97	0.89	0.74–0.96	0.76
MUST	0.6	0.39–0.78	0.97	0.84–0.99	0.94	0.68–0.99	0.78	0.63–0.88	0.57
MST	0.6	0.39–0.78	0.97	0.84–0.99	0.94	0.68–0.99	0.78	0.63–0.88	0.57
MIRT SASKIBD-NR	0.84 0.52	0.63–0.95 0.31–0.72	0.92 0.95	0.77–0.98 0.80–0.99	0.87 0.87	0.66–0.97 0.58–0.98	0.89 0.74	0.74–0.96 0.59–0.85	0.76 0.47
**CD**									
	Sensitivity	95% CI	Specificity	95% CI	PPV	95% CI	NPV	95% CI	Youden index
NS-IBD	0.86	0.58–0.97	0.77	0.58–0.89	0.65	0.40–0.83	0.92	0.73–0.98	0.63
NRS-2002	0.8	0.51–0.94	0.93	0.77–0.98	0.85	0.56–0.97	0.90	0.73–0.97	0.73
MUST	0.53	0.27–0.77	1	0.86–1	1	0.59–1	0.81	0.65–0.91	0.53
MST	0.6	0.32–0.82	1	0.86–1	1	0.62–1	0.83	0.67–0.93	0.53
MIRT SASKIBD-NR	0.8 0.6	0.51–0.94 0.32–0.82	0.93 0.96	0.77–0.98 0.81–0.99	0.85 0.9	0.56–0.97 0.54–0.99	0.90 0.83	0.73–0.97 0.66–0.93	0.73 0.56
**UC**									
	Sensitivity	95% CI	Specificity	95% CI	PPV	95% CI	NPV	95% CI	Youden index
NS-IBD	1	0.65–1	0.5	0.13–0.86	0.76	0.45–0.93	1	0.30–1	0.50
NRS-2002	0.9	0.54–0.99	0.83	0.36–0.99	0.9	0.54–0.99	0.83	0.34–0.99	0.73
MUST	0.7	0.35–0.91	0.83	0.36–0.99	0.87	0.46–0.99	0.62	0.25–0.89	0.53
MST	0.6	0.27–0.86	0.83	0.36–0.99	0.85	0.42–0.99	0.55	0.22–0.84	0.43
MIRT SASKIBD-NR	0.9 0.4	0.54–0.99 0.13–0.72	0.83 0.83	0.36–0.99 0.36–0.99	0.9 0.8	0.54–0.99 0.29–0.98	0.83 0.45	0.36–0.99 0.18–0.75	0.73 0.23

Inflammatory bowel disease (IBD); Crohn’s disease (CD); Ulcerative colitis (UC); IBD Nutritional Screening tool (NS-IBD); Nutritional Risk Screening 2002 (NRS-2002); Malnutrition Universal Screening Tool (MUST); Malnutrition Screening Tool (MST); Malnutrition Inflammation Risk Tool (MIRT); Saskatchewan IBD–Nutrition Risk (SaskIBD-NR).

## Data Availability

Data described in the manuscript, code book, and analytic code will be made available upon request pending.

## Abbreviations

Inflammatory bowel disease (IBD), Crohn’s disease (CD), Ulcerative colitis (UC), Global Leadership Initiative on Malnutrition (GLIM), Bioelectrical impedance vector analysis (BIVA), C-Reactive Protein (CRP), white blood cells (WBC), gastrointestinal (GI), unintended weight loss (UWL), body mass index (BMI), Free Fat Mass (FFM), Free Fat Mass Index (FFMI), Nutritional Risk Screening 2002 (NRS-2002), Malnutrition Universal Screening Tool (MUST), Malnutrition Screening Tool (MST), Malnutrition Inflammation Risk Tool (MIRT), Saskatchewan IBD–Nutrition Risk (SaskIBD-NR), IBD Nutritional Screening tool (NS-IBD), Positive predictive value (PPV), Negative predictive value (NPV), Receiver Operating Characteristic (ROC).
